# Quality of Publicly Available Physical Activity Apps: Review and Content Analysis

**DOI:** 10.2196/mhealth.9069

**Published:** 2018-03-21

**Authors:** Paulina Bondaronek, Ghadah Alkhaldi, April Slee, Fiona L Hamilton, Elizabeth Murray

**Affiliations:** ^1^ eHealth Unit Research Department of Primary Care and Population Health University College London London United Kingdom; ^2^ Community Health Sciences Department College of Applied Medical Sciences King Saud University Riyadh Saudi Arabia; ^3^ Research Department of Primary Care and Population Health University College London London United Kingdom

**Keywords:** exercise, health behavior, mobile applications, health promotion, mHealth, eHealth review

## Abstract

**Background:**

Within the new digital health landscape, the rise of health apps creates novel prospects for health promotion. The market is saturated with apps that aim to increase physical activity (PA). Despite the wide distribution and popularity of PA apps, there are limited data on their effectiveness, user experience, and safety of personal data.

**Objective:**

The purpose of this review and content analysis was to evaluate the quality of the most popular PA apps on the market using health care quality indicators.

**Methods:**

The top-ranked 400 free and paid apps from iTunes and Google Play stores were screened. Apps were included if the primary behavior targeted was PA, targeted users were adults, and the apps had stand-alone functionality. The apps were downloaded on mobile phones and assessed by 2 reviewers against the following quality assessment criteria: (1) users’ data privacy and security, (2) presence of behavior change techniques (BCTs) and quality of the development and evaluation processes, and (3) user ratings and usability.

**Results:**

Out of 400 apps, 156 met the inclusion criteria, of which 65 apps were randomly selected to be downloaded and assessed. Almost 30% apps (19/65) did not have privacy policy. Every app contained at least one BCT, with an average number of 7 and a maximum of 13 BCTs. All but one app had commercial affiliation, 12 consulted an expert, and none reported involving users in the app development. Only 12 of 65 apps had a peer-reviewed study connected to the app. User ratings were high, with only a quarter of the ratings falling below 4 stars. The median usability score was excellent—86.3 out of 100.

**Conclusions:**

Despite the popularity of PA apps available on the commercial market, there were substantial shortcomings in the areas of data safety and likelihood of effectiveness of the apps assessed. The limited quality of the apps may represent a missed opportunity for PA promotion.

## Introduction

### Background

Physical inactivity is an established independent risk factor for a range of serious health conditions including cardiovascular disease, diabetes mellitus, and cancer [1-3]. Physical activity (PA) is also associated with improved mental health [4,5]. The World Health Organization recommends 150 min of moderate or 75 min of vigorous intensity PA per week, yet 31.1% of adults globally fail to achieve this [6]. Behavior change interventions aiming to increase PA tend to have small to moderate effects, with sustainability of intervention effects not well established [7].

Within the new digital health care landscape, the rise of apps creates novel prospects for prevention opportunities and disease management [8]. Mobile health (mHealth) apps, as opposed to traditional face-to-face interventions, are more accessible [9] and provide a range of technology-enhanced features such as accelerometers, visualizations, tailored feedback, and reminders. In addition, recent data show that mobile phone access is now as high among ethnic minority groups in higher income countries as in the rest of the population [10], and the use of mobile phones is increasing steadily in older populations [11], thereby decreasing concerns about the effect of the digital divide on health inequalities. Hence, behavior change interventions delivered using mHealth apps could have the potential to reach a large proportion of the population, thus increasing the public health impact of their small effects [12].

The mHealth app industry has doubled in the last 2 years, with around 165,000 health apps available in the major app stores in 2016 [13]; many of them aiming to increase PA levels. Despite the wide distribution and popularity of health apps, many of them have been rapidly developed [14], and there is lack of evidence of their efficacy. For example, a meta-analysis published by Direito et al [15] found only 7 randomized controlled trials (RCTs) evaluating app intervention for PA and sedentary behavior. It is clearly not feasible for all PA apps to be evaluated by rigorous RCTs, and therefore, alternative methods of evaluating apps are needed. One way of assessing the likely effectiveness of apps is to assess the degree to which they use behavior change theory and adhere to PA guidelines. This research suggests that most PA apps only include a limited number of behavior change techniques (BCTs) [16-18], and they often fail to adhere to PA guidelines [19].

However, quality is about more than effectiveness, although there has been considerable debate about how exactly *app quality* should be defined, with a variety of frameworks available. Recent reviews by BinDhim et al [14] and Bardus et al [20] categorized and evaluated the methods used for quality assessment of apps. Both studies found a considerable variability in methods and measures used to review the quality of health apps. The approaches used to conceptualize and measure quality varied substantially, and the studies tended to focus on either the design quality or on the presence of evidence-based content but not both [20]. The authors called for more research to assess the quality of both design and content of health apps.

Health apps have the potential to be an important health care tool [21]; hence, health care quality indicators were considered appropriate to apply when assessing the quality of the apps. The concept of quality in health care is complex and multifaceted [22]. Maxwell [23] proposed six dimension of health care quality: accessibility (ease of access to all patient groups), relevance to the need of the community, effectiveness, equity (fairness in the distribution), acceptability, efficiency, and economy (desired health outcomes at the lowest cost). On the other hand, Donabedian [24] proposed a different categorization and argued for three crucial elements that pertain to the quality of health care: structure (facilities and health care professionals available), process (actions by which health care is provided), and outcomes (the results of the actions).

The dimensions of quality proposed by Maxwell and Donabedian were developed before the existence of mobile phones and apps and are perhaps more applicable to health care services provided at the point of need, that is, face-to-face. Potential new health care tools apps need a more concise approach, one that  *High quality care for all*:  *NHS Next Stage Review Final Report* [25] appears to provide. This report outlined the 10-year vision for the National Health Service (NHS) with strategies to improve the quality of care. In this report, high-quality health care was defined as being (1) safe, (2) effective, and (3) providing the most positive experience possible. These quality indicators are simple yet comprehensive and sufficiently flexible to apply to potential new health care tools such as PA apps.

### Objective

In this study, we focused on the most popular apps, which we defined as being in the top rankings of the two major app stores. What constitutes the algorithm that determines the app ranking is unknown. However, variables that indicate popularity such as user ratings, volume of ratings and reviews, download and install counts, usage, and uninstalls are likely to contribute to the ranking in the app stores [26]. In addition, potential users are more likely to focus on the top results and rarely examine the search results thoroughly [27]. This method of defining popularity has been used in other studies assessing apps [28-30], and it was selected to gain a representative sample of apps that are most likely to be used and to simulate the user experience of browsing the store to select a health app.

The aim of this study was to assess the quality of publicly available PA apps. Specific objectives were to assess the safety, effectiveness, and provision of the most positive experience in the most popular PA apps.

## Methods

### Study Design

This study is a review and a content analysis of the most popular, publicly available PA apps on the market. *Quality and Risk of Bias Checklist for Studies That Review Smartphone Applications* was used to ensure that methods for apps’ review are adequately described [14].

**Inclusion criteria.**

Apps were included if

- Their main goal was to increase physical activity
- They were targeted at healthy adults
- They had stand-alone functionality

**Exclusion criteria.**

Apps were excluded if

- The app focused on multiple behaviors, as it would have been difficult to isolate the content pertaining to physical activity
- The target population was patients with a specific health condition, as these users were likely to have different needs to healthy adults
- They were sold as part of a pack (“bundle”), as it would not have been possible to assess the popularity of the individual apps in this bundle

### Sample Identification

A sample of top-ranked 400 PA apps was obtained from the UK’s versions of the iTunes and Google Play stores on October 17, 2016. As previous research indicated an association between price and inclusion of BCTs [18,31,32], both free and paid apps were included in the study. Apps’ titles and descriptions from the “Health and Fitness” category in both stores (100 iTunes free + 100 iTunes paid + 100 Google Play free + 100 Google Play paid) were screened against the inclusion and exclusion criteria. (Textboxes 1 and 2)

### Sample Assessment

From the apps identified, 65 were randomly selected for the assessment using the random number generator function in Excel (Microsoft). As the largest subset of health apps on the market (30%) [13] target PA, it was expected that a high number of apps would fulfil the inclusion criteria. We were undertaking a parallel study to assess the association between quality indicators and user rating, and the choice of n=65 was based on the power calculation for that parallel study.

The apps were downloaded onto an iPhone SE and 6 (running iPhone operating system [iOS, Apple Inc] 10.2.1 and 9.3.4 software, respectively) and Android Samsung Galaxy S6 and J5 (running 6.0.1 or 5.1.1 software, respectively) and assessed using a pro forma evaluation. Each app was left running in the background for 2 days for the assessors to explore any reminders or notifications. If two apps were identified as duplicates and there appeared to be consistency of design and content between both operating systems, the apps were assessed on an iPhone only. The sample identification and assessment was conducted independently by two reviewers (PB and GA), and any discrepancies were resolved through discussion.

### Data Extraction

#### Descriptive Data

We extracted the following descriptive data from both app stores: app’s name, brief description, type of PA targeted (eg, running, walking, and whole body workout), platform on which the app was available, developer’s name, rank, number of ratings, cost, size, last update, and version.

#### Application of Health Care Quality Indicators to Physical Activity Apps

The methods of operationalizing the three quality indicators of safety, effectiveness, and provision of the most positive experience possible for the selected apps is described below.

##### Safety of Physical Activity Apps

For the safety indicator of health apps, privacy and security of users’ data were considered. The privacy and security assessment was based on the recommendations of the Information Commissioners Office [33] and Online Trust Alliance [34]. It comprises of 8 questions evaluating the availability, accessibility of privacy policy, data gathering and sharing practices, and data security as is discussed in the privacy statement (see Multimedia Appendix 1 for data privacy and security assessment).

##### Likelihood of Effectiveness of Physical Activity Apps

As research on PA app efficacy is lacking, the likelihood of effectiveness was assessed by quantifying the presence of BCTs. Furthermore, many quality assessment procedures include an evaluation of the intervention development processes [35,36]. For example, involving key stakeholders in the development process is important to produce an intervention that meets user needs and increases the likelihood of intervention implementation [37]. Hence, data on the organizational affiliation of the developer, as well as expert and user involvement in the development process was collected. In addition, any evidence of scientific evaluation was also extracted.

###### Behavior Change Techniques

The BCT taxonomy v1 [38] was used to assess the number of BCTs in each app and the frequency of each BCT in the app sample overall. The coding manual provides guidelines to investigate the presence of 93 BCTs in behavior change interventions and has been used in previous studies that aimed to characterize BCTs in health apps [16,28,39-41]. In line with the instructions, we coded each BCT as Absent, Present + (BCT present in all probability but evidence unclear), and Present ++ (BCT present beyond all reasonably doubt).

**Table 1:** The application of the health care quality indicators to physical activity apps.

Quality indicator of health care	Applying the indicator to health apps
Safety	Privacy and security of data
Effectiveness	Behavior change techniques (Michie et al [38])
	Development and evaluation process: Organizational affiliation; Expert involvement; User involvement; and Evidence of scientific evaluation
Positive experience	User ratings
	Usability

###### Quality of Development Process and Evidence for Evaluation

The evaluation of the quality of development process was based on the information provided in the app stores, the app website (if existent), and within the app itself. The following characteristics of the app content development were extracted: organizational affiliation (university, medical, government, or other nonprofit institutions); expert involvement (eg, fitness expert, behavior change specialist, and medical professional); and evidence for user involvement in the development of an app. The evidence for app evaluation was assessed by searching the name of the app in the following scientific databases: PubMed, ACM Digital Library, IEEE Xplore, and Google Scholar.

##### Provision of the Most Positive Experience in Physical Activity Apps

The provision of the most positive experience was operationalized using (1) the user ratings in app stores and (2) through formal usability assessment conducted by the two reviewers using the System Usability Scale (SUS) [42]. The average star rating (range: 1-5 stars) was calculated by summing the number of stars and dividing them by the number of users who submitted ratings. SUS is a valid and reliable measure of overall usability (from 0-100) and consists of 10 items that are ranked on a 5-point Likert scale, from *strongly disagree* to *strongly agree*. The wording of the 8th statement was changed from *cumbersome* to *awkward* as recommended [43-45]. Second, the word *system* was replaced by *app* to make the scale applicable to the sample in this study. The interpretation of the SUS score used the thresholds proposed and validated by Bangor et al [43].

### Summary of Application of Quality Indicators

The application of health care quality indicators to apps is summarized in Table 1.

#### Interrater Reliability

Interrater reliability for the presence or absence of the BCTs was ascertained by calculating Cohen kappa statistic [46] for each item. In addition, prevalence-adjusted bias-adjusted kappa (PABAK) [47] was assessed for the presence or absence of BCTs. The occurrence of high prevalence of negative agreement (when both rates agree that the BCT is absent) is very likely in the context of inclusion of BCTs in an app. When high prevalence of the identical response is seen, the kappa value results in low proportion of agreement, although the observed agreement is high [48]. The a priori strategy for assessing the sample was to complete the extraction of data for 10 apps to resolve any discrepancies in understanding of the measures before extracting the rest of data. Hence, the interrater reliability was assessed on 55 apps.

#### Statistical Analysis

The number of BCTs in the apps was summarized using the mean, standard deviation, median, 25^th^ and 75th percentiles, and the maximum and minimum. Similar statistics were used to summarize user ratings, cost, size, and SUS score. Proportions were used to summarize the variables: data privacy and security, organization affiliation, expert and user involvement, and the evidence of evaluation in peer-reviewed journals.

The summary descriptive tables were presented for each store for free and paid apps separately and in total as app stores have separate rankings based on the cost. To assess if there was a difference in store characteristics between free and paid apps, *t* tests were used to compare the average user ratings, size, and the number of BCTs; Wilcoxon test was used to compare the number of ratings; and Fisher exact was used for last update (<3 months, 3-6 months, and >6 months), organizational affiliation, expert and user involvement, and presence of any peer-reviewed studies.

## Results

### Sample Identification

**Figure 1:**
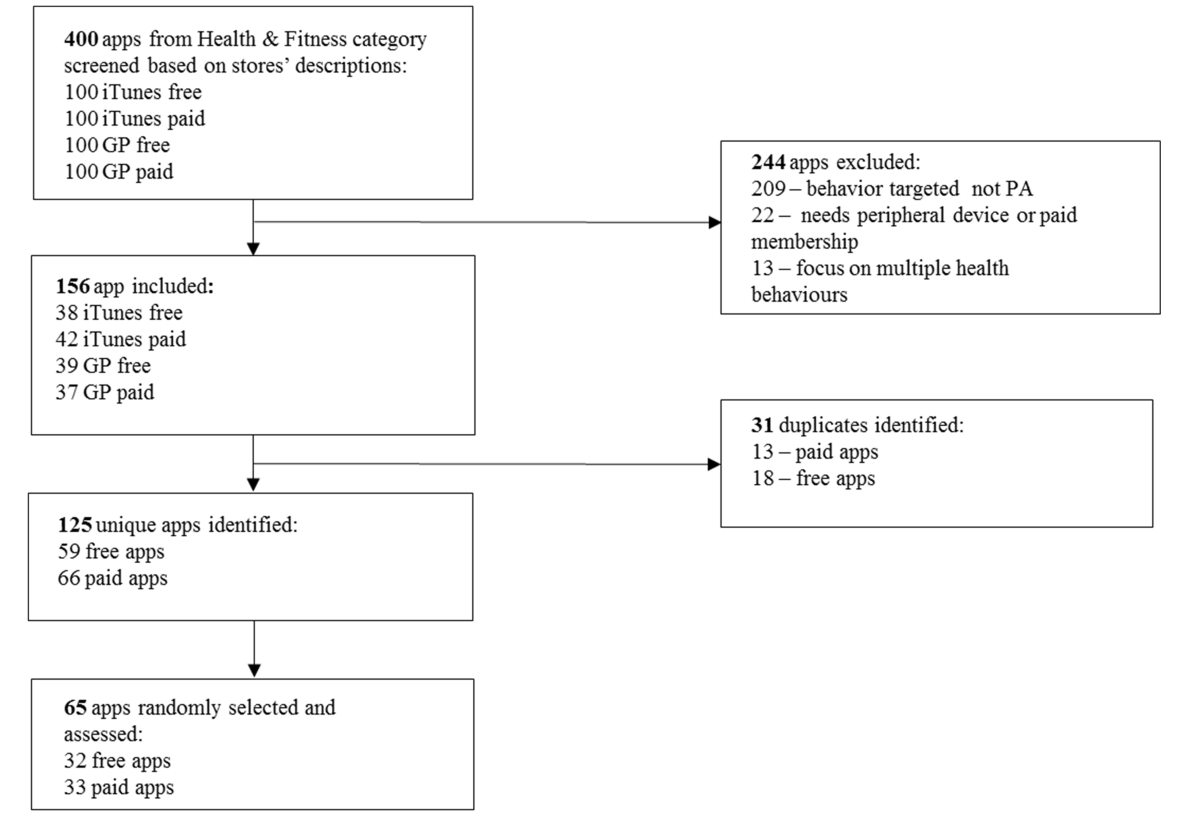
Flowchart of the apps included in the analysis. PA: physical activity.

Out of 400 apps, 244 apps were excluded (209 apps did not target PA, 22 apps needed a peripheral device or paid membership to use the app, and 13 apps focused on multiple health behaviors), and 156 met the inclusion criteria (see Figure 1). A total of 31 duplicates were found. Subsequently, a sample of 125 unique apps was identified. A total of 65 apps, 32 free and 33 paid, were assessed.

### Sample Characteristics

Descriptive data for the app sample are presented in Tables 2 and 3, whereas the data for each app separately is presented in Multimedia Appendix 2. There were no statistically significant differences in the number of ratings, cost, size, and last update between the free and paid apps in either iTunes or Google Play store.

**Table 2:** Descriptive data for iTunes store.

Descriptive data for iTunes		Free—iTunes (N=21)	Paid—iTunes (N=24)	Total—iTunes (N=45)	*P* value
**Number of ratings **					
	Mean (SD)	3408.4 (5848.4)	773.7 (1187.0)	2031.2 (4289.7)	.49
	Median	758	127	550	
	25-75 percentile	438.0-3698.0	47.0-1247.0	85.5-1719.0	
	Min-max	14-24530	11-3845	11-24530	
**Cost—iTunes (GBP^a^)**					
	Mean (SD)	N/A^b^	2.5 (1.5)	N/A	
	Median	N/A	2.3	N/A	
	25-75 percentile	N/A	1.5-3.0	N/A	
	Min-max	N/A	1-8	N/A	
**Size of app (megabyte)**					
	Mean (SD)	88.4 (49.8)	94.9 (75.4)	91.8 (64.1)	.74
	Median	74.3	83.3	82.2	
	25-75 percentile	52.0-131.0	61.7-102.0	58.1-104.0	
	Min-max	11-164	9-376	9-376	
**Last update**					
	<3 months, n (%)	13 (61.9)	7 (29.2)	20 (44.4)	.09
	3-6 months, n (%)	3 (14.3)	7 (29.2)	10 (22.2)	

^a^GBP: British pound.

^b^N/A: not applicable.

**Table 3:** Descriptive data for Google Play store.

Descriptive data for Google Play		Free—Google Play (N=21)	Paid—Google Play (N=16)	Total—Google Play (N=37)	*P* value
**Number of ratings**					
	Mean (SD)	119000.7 (165085.0)	14457.9 (43700.8)	73793.0 (136723.2)	>.99
	Median	44923	1720.5	5856	
	25-75 percentile	5827.0-199596.0	384.5-6452.0	1475.0-78204.0	
	Min-max	206-625077	7-177277	7-625077	
**Cost—Google Play (GBP^a^)**					
	Mean (SD)	N/A^b^	3.6 (2.3)	N/A	
	Median	N/A	2.7	N/A	
	25-75 percentile	N/A	2.3-5.0	N/A	
	Min-max	N/A	1-9	N/A	
**Size of app (megabyte)**					
	Mean (SD)	28.4 (21.2)	43.4 (34.2)	34.9 (28.2)	.11
	Median	26.8	31.5	29.6	
	25-75 percentile	12.2-38.5	27.7-54.0	15.4-43.9	
	Min-max	2-73	1-145	1-145	
**Last update**					
	<3 months, n (%)	16 (76)	7 (44)	23 (62)	.12
	3-6 months, n (%)	1 (5)	3 (19)	4 (11)	
	>6 months, n (%)	4 (19)	6 (38)	10 (27)	

^a^GBP: British pound.

^b^N/A: not applicable.

The apps were categorized into five groups according to their primary focus. These were as follows: workout apps that demonstrate various exercises (31/65, 47%), tracking of movement apps that provide mapping of the running or walking or cycling routes (13/65, 20%), running programs that have prespecified goals reached by incremental increase in run-to-walk ratio (12/65, 18%), pedometers-based apps that count steps (6/65, 9%), and interval timers that enable the user to time their work or rest period (3/65, 4%).

### Data Privacy and Security

#### Availability and Accessibility of Privacy Policy

The privacy policy was available for 46 (70%, 46/65) apps overall. In one case, the link to the privacy policy was provided but did not work, and the app was indicated as not having a privacy policy. Of those that had privacy policy, only 4 (8%, 4/46) apps had a short form privacy and security notice that highlighted key data practices that were disclosed in detail in the full privacy policy (see Table 4). There were nine instances where the short form notice was not applicable because of the policy already being concise. Multilingual policies were rare, with only 5 apps having a policy in another language. Apps that were developed outside the United Kingdom were more likely to provide multilingual policies.

#### Data Gathering and Sharing

Most of the apps (80%) reported collecting personally identifiable information. In one instance, the developer did not discuss the data gathering practices. In 34 instances (80%, 34/46), the developers stated that they share the data they gather with 3rd parties. There were two instances where the developer did not discuss data sharing practices. In many cases, the policies stated that “data shall not be shared, except for” followed by a list of exceptions that were vague and general. In these instances, the reviewers considered that the data were shared by the 3rd party.

#### Data Security

Only 41% (19/46) of the apps described how the users’ data were protected. The privacy policies stated that data safety is important to their practices but did not provide information on how data security was ensured.

### The Presence of Behavior Change Techniques

There was “almost perfect” agreement between the reviewers for the coding of BCT presence or absence: PABAK=0.94, 95% CI 0.93-0.95, kappa=.78 (“substantial”), 95% CI 0.75-0.81.

**Table 4:** Data gathering, sharing and security as described in the privacy policy (within those that had the policy, N=46). Note: 29% (19/65) did not have a privacy policy available.

Data gathering, sharing, and security as described in the privacy policy		Free (N=24), n (%)	Paid (N=22), n (%)	Total (N=46), n (%)
**Is the privacy policy available without the need to download the app?**				
	Yes	24 (100)	22 (100)	46 (100)
**Is the privacy policy available within the app?**				
	No	13 (44)	16 (55)	29 (63)
	Yes	11 (64)	6 (35)	17 (36)
**Is there a short form notice (in plain English) highlighting key data practices?**				
	No	17 (70)	16 (72)	33 (71)
	Yes	4 (16)	0 (0)	4 (8)
	Not applicable	3 (12)	6 (27)	9 (19)
**Is the privacy policy available in any other languages?**				
	No	20 (83)	21 (95)	41 (89)
	Yes	4 (16)	1 (4)	5 (10)
**Does the app collect personally identifiable information?**				
	No	2 (8)	6 (27)	8 (17)
	Yes	21 (87)	16 (72)	37 (80)
	Not specified	1 (4)	0 (0)	1 (2)
**Does the app share users’ data with a 3rd party?**				
	No	2 (8)	8 (36)	10 (22)
	Yes	21 (87)	13 (59)	34 (74)
	Not specified	1 (4)	1 (4)	2 (4)
**Does the app say how the users' data security is ensured? For example, encryption, authentication, and firewall**				
	No	13 (54)	14 (63)	27 (58)
	Yes	11 (45)	8 (36)	19 (41)

**Table 5:** Descriptive statistics for the inclusion of the behavior change techniques (BCTs).

Inclusion of the BCTs		Free (N=32)	Paid (N=33)	Total (N=65)	*P* value
**Total BCTs**					
	Mean (SD)	6.6 (3.0)	7.5 (2.9)	7.0 (2.9)	.21
	Median	7	8	8	
	25-75 percentile	5.0-8.0	6.0-10.0	5.0-9.0	
	Min-max	1-12	1-13	1-13	

The total number of BCTs for free and paid apps sample was similar (see Table 5). Every app contained at least one BCT, and the maximum number of BCTs was 12 for free and 13 for paid apps. The median number of BCTs was 7 for free and 8 for paid apps (see Multimedia Appendix 3 for the graph of the distribution of the BCTs in apps).

**Figure 2:**
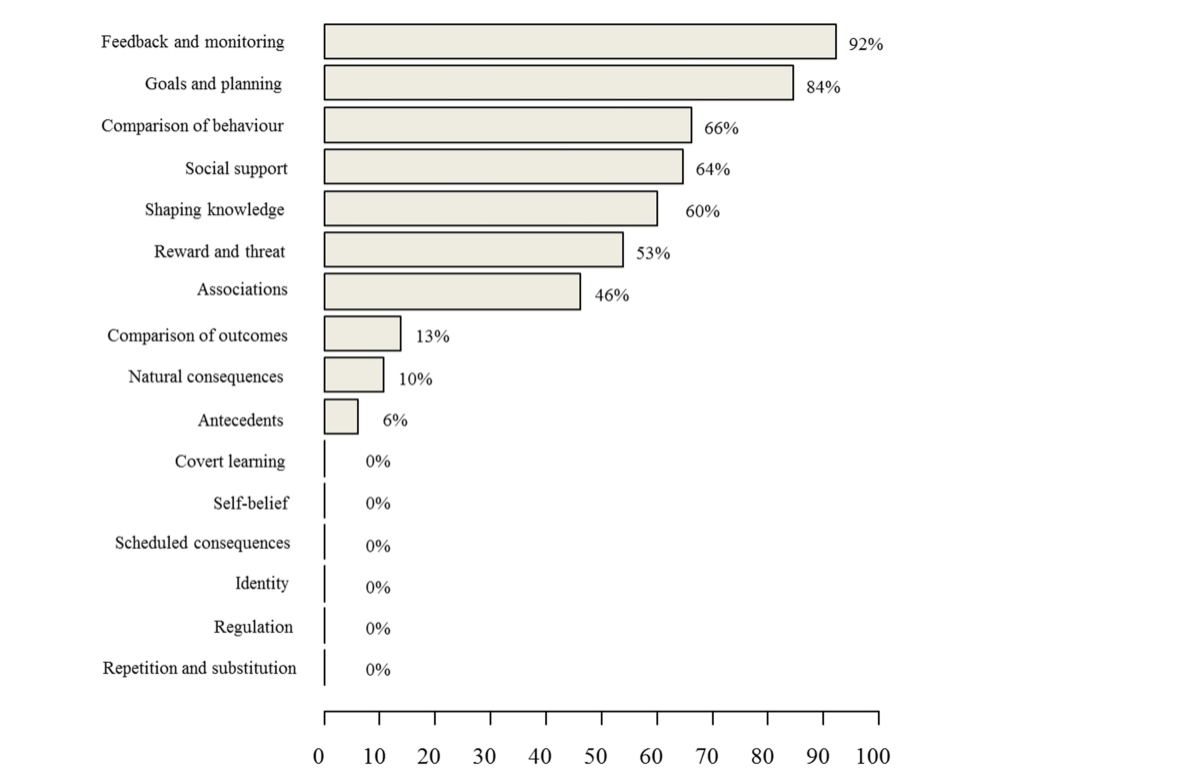
Frequency of behavior change techniques (BCTs) incorporated by physical activity (PA) apps, presented by BCT groups.

**Figure 3:**
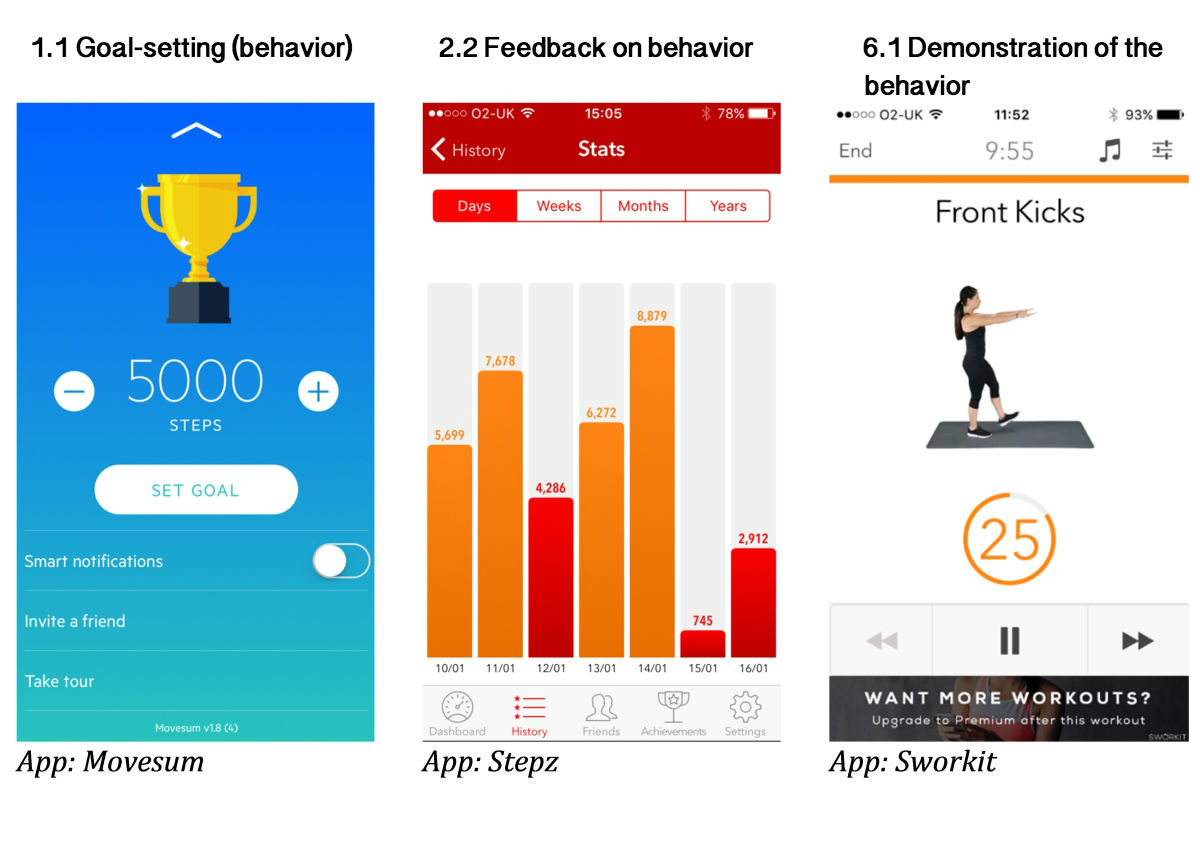
Examples of the most common behavior change techniques (BCTs) from the most frequent BCT groups: (1) goals and planning: 1.1 Goal setting (behavior), (2) feedback and monitoring: 2.2 Feedback on behavior, and (3) comparison of behavior: 6.1 Demonstration of the behavior.

Figure 2 shows the frequency of the common BCT groups. The “Feedback and monitoring” group was the most common, with 92.3% of apps containing at least one BCT of this group, most commonly “Feedback on behavior” and “Feedback on outcome(s) of behavior” BTCs. “Goals and planning” (“Goal setting” and “Action planning” BCTs) were also well represented at 84.6%. More than half of the apps included BCTs from the “Comparison of behavior” group (66.2%), which most likely was “Demonstration on the behavior” (see Figure 3 for the examples of the app features that included BCTs from the most common BCT groups). “Social support” (64.6%), “Shaping knowledge” groups (60%), and “Associations” (46.2%) were common, but only one BCT from each of these groups were present. “Reward and threat” group (53.8%) was common with two BCTs only (“Social reward” and “Nonspecific incentive”). Other BCT groups were rare: less than 15% of apps contained BCTs from the “Comparison of outcomes” group; “Natural consequences” and “Antecedents” represented 10.8% and 6.2% of the total BCTs, respectively. The remaining BCT groups were nonexistent in the PA apps. Multimedia Appendix 4 presents the frequency of individual BCTs within the groups’ BCTs (BCTs that occurred in at least five apps are shown).

### Quality of App Development and Evaluation Process

Only 1 app had a noncommercial affiliation, *One You Couch to 5K*, which was developed by Public Health England (see Table 6). None of the apps reported user involvement during development. Twelve out of 65 apps (4 free and 8 paid) consulted with experts to design the content of the app. Nine out of 23 free apps (28.1%) had a study associated with the apps published in a peer-reviewed journal. In comparison, for only 3 paid apps (9.1%), there was a peer-reviewed study found.

### Positive Experience

#### User Ratings

The median user rating in iTunes was 4.4 and 4.5 in Google Play and did not differ between free and paid apps in either stores (see Table 7).

**Figure 4:**
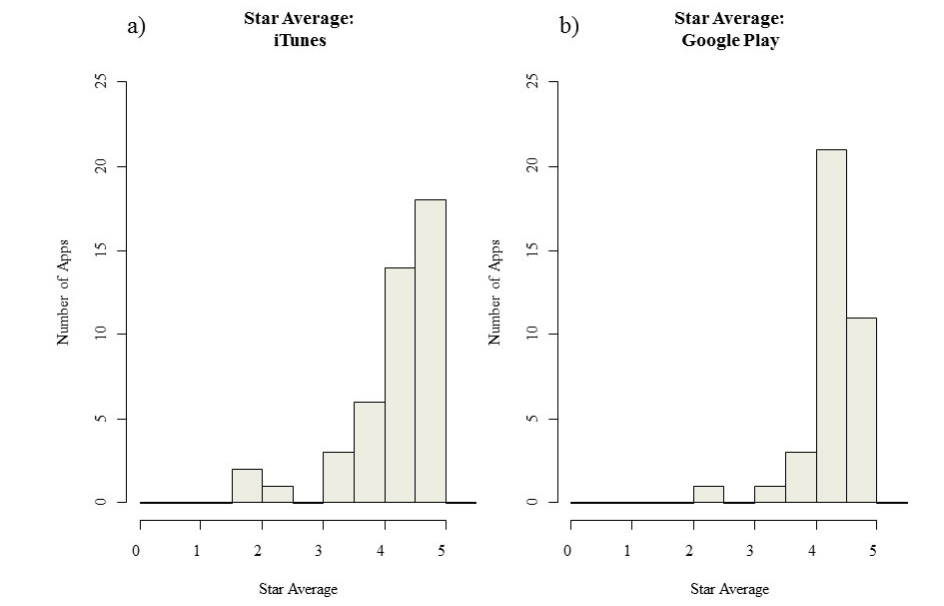
Distribution of user ratings in iTunes and Google Play.

In both stores, the 25th percentile was around 4 stars (4.0 in iTunes and 4.4 in Google Play), suggesting that the user ratings tended to be high, and only 25% of ratings were below 4 stars. The histograms of star ratings in both stores (Figure 4) showed the skewness of the star average distribution.

#### Usability

The average SUS score for the apps was similar for both free and paid apps, with median of 86.3 (see Table 8). Using the descriptors suggested by Bangor et al [43], the score can be described as “excellent.” Fifty percent of the total average SUS score fell between 75.0 and 92.5, and 25% had a score higher than 92.5, suggesting that more than 75% of the app sample assessed could be described as having “good” to “excellent” usability. See Multimedia Appendix 5 for the graph of the distribution of the SUS score averaged between the two reviewers.

**Table 6:** Descriptive data for the quality of app development and evaluation process: organizational affiliation, expert and user involvement, and evidence of evaluation in peer-reviewed journals.

The quality of app development and evaluation process		Free (N=32), n (%)	Paid (N=33), n (%)	Total (N=65), n (%)	*P* value
**Any affiliation**					
	Commercial	31 (96)	33 (100)	64 (98)	.49
	Government institution	1 (3)	0 (0)	1 (1)	
**Any expert**					
	No	28 (87)	25 (75)	53 (81)	.34
	Yes	4 (12)	8 (24)	12 (18)	
**Any user involvement**					
	No	32 (100)	33 (100)	65 (100)	
**Any peer journal**					
	No	23 (71)	30 (90)	53 (81)	.06
	Yes	9 (28)	3 (9)	12 (18)	

**Table 7:** Descriptive statistics for user ratings (1-5 stars) in iTunes and Google Play.

User ratings		Free	Paid	Total	*P* value
**iTunes**		(N=21)	(N=24)	(N=45)	
	Mean (SD)	4.1 (0.8)	4.3 (0.6)	4.2 (0.7)	.22
	Median	4.4	4.6	4.4	
	25-75 percentile	4.0-4.6	4.0-4.8	4.0-4.6	
	Min-max	2-5	3-5	2-5	
**Google Play**		(N=21)	(N=16)	(N=37)	
	Mean (SD)	4.4 (0.5)	4.4 (0.3)	4.4 (0.4)	.90
	Median	4.5	4.5	4.5	
	25-75 percentile	4.4-4.6	4.4-4.6	4.4-4.6	
	Min-max	2-5	4-5	2-5	

**Table 8:** Descriptive data for the System Usability Scale (SUS) assessment.

Usability assessment		Free (N=32)	Paid (N=33)	Total (N=65)	*P* value
**SUS score**					.17
	Mean (SD)	81.3 (12.6)	85.5 (11.9)	83.4 (12.4)	
	Median	85	87.5	86.3	
	25-75 percentile	71.9-91.3	80.0-93.8	75.0-92.5	
	Min-max	53-100	58-100	53-100	

## Discussion

### Principal Findings

This study described the most popular PA apps on the market, focusing on the quality determinants of safety (data privacy and security), effectiveness (BCTs and development and evaluation quality), and provision of the most positive experience possible (user ratings and usability). Overall, our findings suggest that most of the apps in this sample were of reasonable quality in terms of the user experience, but there were substantial shortcomings in the areas of safety and effectiveness. The assessment of data privacy and security showed that the privacy policy was not available for 29.2% of the apps. Most apps collected personally identifiable information, shared users’ data with a third party, and more than half of the apps did not specify how they ensure data security. Every app contained at least one BCT, with an average of 7. The maximum number of BCTs was 13, and the most common BCTs related to provision of feedback on behavior. All but one app had commercial affiliation, 12 consulted an expert, and none reported involving users in the app development. Only 12 of 65 apps had a peer-reviewed study connected to the app but only one app was assessed for efficacy in a trial [49]. User ratings were high, with only a quarter of the ratings falling below 4 stars. Similarly, the usability scores were “good” to “excellent.” There was no statistically significant difference between free and paid apps on the characteristics or quality indicators.

### Safety of Apps

The assessment of privacy policy showed that privacy and security of users’ data could be substantially improved. Our results are consistent with previous studies assessing data safety. Huckvale et al [8], who assessed the apps from the NHS Apps Library, found that 20% of apps did not have privacy policy, and most of the apps breached users’ data privacy and security. Collecting and analyzing consumer data by app developers can have advantages for the users, such as personalization and improvement of the products [35]. However, the information about these practices ought to be transparent and understandable [36] to enable the potential user to make an informed decision to download the app. Regulatory oversight concerning data protection is challenging because of the large scale of the app market. In consequence, ensuring the privacy and security of data is left in the hands of app developers [50].

### Likelihood of Effectiveness

The apps in the review contained, on average, 7 BCTs. The results of this study are similar to those found in previous reviews of PA apps: Middelweerd et al [17] found that, on average, 5 BCTs were used in each app; Conroy et al [16] reported between 1 and 13 BCTs with a mean of 4.2; and a study using the same BCT taxonomy as the one in this study found, on average, 6.6 BCTs [18].

The most common BCTs were feedback and monitoring, goal setting, and action planning. These self-regulation strategies have been shown to be effective in increasing PA behavior [51,52]. However, the BCTs from 9 out of 16 BCT groups were rare or nonexistent in the apps assessed, and the BCTs that were present constituted 14% of the current BCT taxonomy.

The effect of the number of BCTs on efficacy of the interventions remains inconclusive. Although there is some evidence that higher number of BCTs produces larger effect sizes in Web-based interventions [53], others show no effect [51]. The evidence of what BCTs are most likely to increase the likelihood of behavior change is unknown. It is possible that certain BCTs are more efficacious when present together producing a synergistic effect [54]. The use of variety of BCTs groups, as well as the techniques within the BCT group, would theoretically increase effectiveness by addressing various barriers to PA. For example, within the “Goals and planning” BCT group, only 3 out of 9 BCTs were utilized. Implementing features that utilize other BCTs that enable goal setting and planning (eg, problem-solving technique, asking the user to commit to their goal, and providing an opportunity for the user to review their goal) might increase the likelihood of effectiveness of the app.

The use of evidence and theoretical frameworks is vital in developing behavior change interventions [55]. The COM-B (capability, opportunity, motivation, and *behavior*) model of behavior change [56] enables developers to systematically identify the barriers and facilitators of the behavior targeted and to select intervention components that will address these barriers to increase the likelihood of behavior change.

The results suggest that the quality of the app development and evaluation process could be improved. We did not find any evidence of user involvement, and most apps were commercially developed with the rare involvement of experts. Similar results were found in previous reviews [28,57], and there is evidence to suggest that expert involvement predict the number of app download [58]. Indeed, the user-centered design framework stresses the importance of understanding the contextual experiences of potential users, as well as inclusion of multidisciplinary skills and perspectives when developing products and services. Our results also support previous research showing the lack of evidence for scientific evaluation of the apps on the market [59,60]. We found only 12 studies in peer-reviewed journals that were associated with the apps. However, only one app was used in a pragmatic RCT [49], and the study was not conducted by the app developer.

### Positive Experience

The usability of the apps reviewed was high. Likewise, user ratings of the PA apps were high, with only a quarter of the ratings receiving less than 4 stars. Similarly, Mendiola et al [61] found that usability was related to user ratings in a general sample of health apps. The competition for customer in the app stores is high, with 90% of apps in the app stores not attracting enough attention to feature in the ranking of the app stores and consequently not visible for the user, called “App Zombies” [62]. High-quality graphic design, visual appeal, and ease of use are more likely to attract potential customers to download and engage with the app. However, it is unknown whether these variables relate to effectiveness of the apps. There is evidence to suggest that Web-based interventions with higher usability tend to be more effective [54]. However, continued engagement with an app may suggest engagement with the intervention or unhealthy dependence [63].

### Strengths

The strengths of this study include a systematic approach to sample identification and assessment. First, the sample of apps was identified by screening 400 apps in two major app distribution platforms, including both free and paid apps. Second, the sample was identified and assessed by 2 independent reviewers. Third, the assessment tools covered various aspects of quality, both inclusion of theory as well as user experience using subjective (user ratings) and objective (usability) measures.

### Limitations

First, it is unknown what variables are included in the ranking algorithm of the top apps from which the sample was selected. It is likely that usage data and user ratings comprise the ranking [26], but other unknown variables may also be included. Second, the possibility that user ratings were influenced by fake reviews cannot be excluded. [64,65]. However, there is a reliance on genuine users of the app to mark it down if the app does not live to their expectations, and this review included popular apps with high number of ratings (2.8 million). Third, data privacy and security assessment was limited to the analysis of the policy. There is evidence of inconsistency between the policy statement and the actual practices of app developers [8]. Fourth, the quality of app development process was based on the information provided in the app stores, the app website, and within the app itself; hence, it is possible that some data were missed if they were not available on the Web. Finally, the evidence for app evaluation was assessed by searching the name of the app in the popular scientific databases. If the name of the app was absent in the title or abstract, then the relevant paper would not have been found.

### Implications

More studies are needed to assess what predicts higher user rating. It is unknown what features or characteristics of apps users like and perceive to be effective in increasing their PA. It is possible that there is a discrepancy between what is liked and what is more likely to be effective. Second, research is needed to understand the use of PA apps to design effective digital tools. There is little knowledge concerning how users adopt these apps into their routines and what are the facilitators and barriers to increasing PA using apps. Third, the optimal number of BCTs in PA app remains unknown. It is likely that different BCTs may be more suitable for different modes of delivery (face-to-face, Web-based, and app), For example, social support might produce better results when delivered face-to-face rather than via an app. Alternatively, automatic monitoring and feedback on PA in apps can facilitate self-regulation and may be considered as a more efficient method than self-monitoring using diaries.

Although popularity of the apps is high, health care professionals and potential users need to be aware of the limitation in the safety of personal data, as well as the limitation in the quality of the apps to change behavior. Currently, it is not possible to recommend apps that are most effective, but attempts to create a database of high-quality apps are in progress. For example, the National Information Board is developing an app accreditation model that consists of a 4-stage assessment framework that aims to establish a database of high-quality health apps [66].

### Conclusions

This study examined the quality of the most popular PA apps currently available on the market. Although usability and user ratings of app were high, there was a concerning lack of safety controls for users’ personal data for the majority of the apps, the apps included limited number of BCTs that mostly related to feedback on behavior, and the quality of the content and development processes were suboptimal. The technological development and the potential for profit far outpaced the research on the ability of these apps to support PA behavior change. With 165,000 apps on the market, this represents a loss of opportunity for health promotion on a large scale.

## Appendix

Multimedia Appendix 1 Data privacy and security assessment based in the content of privacy policy.

Multimedia Appendix 2 Individual-level data for the sample of apps assessed.

Multimedia Appendix 3 Graph of the distribution of the BCTs in PA apps.

Multimedia Appendix 4 Frequency of individual BCTs within the groups BCTs (BCTs that occurred in at least five apps are shown).

Multimedia Appendix 5 Graph of the distribution of the SUS score averaged between the two reviewers.

## Abbreviations

BCT: behavior change technique
mHealth: mobile health
NHS: National Health Service
PA: physical activity
PABAK: prevalence-adjusted bias-adjusted kappa
RCT: randomized controlled trial
SUS: System Usability Scale
