# Supplementary material for: Quality of Publicly Available Physical Activity Apps: Review and Content Analysis
Source: JMIR Mhealth Uhealth. 2018 Mar 21;6(3):e53. doi: 10.2196/mhealth.9069 (PMC5885062; doi:10.2196/mhealth.9069)
Supplement: Multimedia Appendix 1 [file mhealth_v6i3e53_app1.pdf]

Data privacy and security assessment

Q1: Availability	Is there privacy information available? (only continue if answered 'Yes')	Yes/No
Q2: Availability	Is the privacy information available without the need to download the app? (example: app store, via a link to the privacy policy or the app's website)	Yes/No
Q3: Availability	Is the privacy information available within the app?	Yes/No
Q4: Accessibility	Is there a short form notice (in plain English) highlighting key data practices which are disclosed in detail in the full privacy policy?	Yes/No/NA[a]
Q5: Accessibility	Is the privacy policy available in any other languages?	Yes/No
Q6: Data gathering	Does the app collect Personally Identifiable Information?	Yes/No/NS
Q7: Data sharing	Does the app share users' data with 3rd party?	Yes/No/NS
Q8: Data security	Does the app say how the users' data security is ensured? e.g. encryption, authentication, firewall system	Yes/No

[a] ONLY indicate NA if the policy is already short
