# Supplementary material for: Quality of Publicly Available Physical Activity Apps: Review and Content Analysis
Source: JMIR Mhealth Uhealth. 2018 Mar 21;6(3):e53. doi: 10.2196/mhealth.9069 (PMC5885062; doi:10.2196/mhealth.9069)
Supplement: Multimedia Appendix 2 [file mhealth_v6i3e53_app2.pdf]

Individual-level data for the sample of apps assessed

Name	Type of PA app	Number of ratings (iTunes / GP)	Cost (iTunes / GP)	Size in MB (iTunes / GP)	Last update (iTunes / GP)	Have privacy policy	Available without app download	Available after app download	Short form notice	Other languages	Collects PII	Shares with 3rd party	Total number of BCTs	Expert involvement	Any studies associated with the app	Avg. user rating (1-5 stars) (iTunes / GP)	SUS
Fitbit	TR	7871/ 207000	FREE	104 / 22.35	09/12/2016 - 15/12/2016	●	●	●	●	○	●	●	10	○	●	3.9 / 4	66
Strava Running and Cycling GPS	TR	10742 / 199596	FREE	59.2 / 21.41	09/01/2017 - 03/01/2017	●	●	●	●	○	●	●	8	○	●	4.6 / 4.5	60
Pacer – Pedometer plus Weight Loss and BMI Tracker	P	4484 / 250669	FREE	85.4 / 9.64	12/01/2017 - 09/01/2017	●	●	●	●	○	●	●	10	○	●	4.6 / 4.5	75
Map My Run – GPS Running & Workout Tracker	TR	24530 / 135087	FREE	164 / 70.02	22/12/2016 - 22/12/2016	●	●	●	○	○	●	●	8	○	●	4.5 / 4.5	85
Adidas train & run	TR	NA / 78868	FREE	NA / 72.53	NA - 30/11/2016	●	●	●	●	●	●	●	10	○	○	NA / 4.3	76
Steps Pedometer & Step Counter Activity Tracker	P	438 / 0	FREE	25.7 /NA	30/11/2015 - 07/12/2016	○							3	○	○	4.1 / NA	94

Name	Type of PA app	Number of ratings (iTunes / GP)	Cost (iTunes / GP)	Size in MB (iTunes / GP)	Last update (iTunes / GP)	Have privacy policy	Available without app download	Available after app download	Short form notice	Other languages	Collects PII	Shares with 3rd party	Total number of BCTs	Expert involvement	Any studies associated with the app	Avg. user rating (1-5 stars) (iTunes / GP)	SUS
7 Minute Workout by Simple Design Ltd	W	NA / 351356	FREE	NA/ 13.67	19/12/2016 - 19/12/2016	●	●	●	NA	○	○	○	11	○	○	NA / 4.5	55
Runtastic Running & Fitness	TR	1760 / 625077	FREE	144 / 33.08	NA - 13/12/2016	●	●	○	○	○	○	○	8	○	●	4.5 / 4.5	53
Home workout MMA Spartan Free	W	NA / 9913	FREE	NA/ 28.41	12/01/2017 - NA	○							5	○	○	NA / 4.6	91
Stepz – Pedometer & Step Counter	P	387 / 0	FREE	43.4 /NA	27/05/2016 - NA	●	●	○	○	●	●	●	8	○	○	4.6 / NA	100
Interval Timer – Timing for HIIT Training and	IT	119 / 0	FREE	14.3 /NA	17/01/2017 - NA	○							2	○	○	4.4 / NA	85
Sworkit – Custom Workouts for Exercise & Fitness	W	1383/ 0	FREE	151 /NA	NA - 20/10/2016	●	●	●	○	○	●	●	8	●	●	4.7 / NA	96

**Name**	Type of PA app	Number of ratings (iTunes / GP)	Cost (iTunes / GP)	Size in MB (iTunes / GP)	Last update (iTunes / GP)	Have privacy policy	Available without app download	Available after app download	Short form notice	Other languages	Collects PII	Shares with 3rd party	Total number of BCTs	Expert involvement	Any studies associated with the app	Avg. user rating (1-5 stars) (iTunes / GP)	SUS
Fitness & Bodybuilding	W	NA / 44923	FREE	NA/ 31.5	29/01/2016 - NA	●	●	●	○	○	●	●	7	●	○	NA / 4.6	80
Daily Workouts FREE	W	826 / 0	FREE	52 /NA	03/11/2016 - 21/06/2016	●	●	●	○	○	●	●	7	●	○	4.6 / NA	71
30 Day Ab Challenge FREE	W	498 / 996	FREE	67.7 / 6.72	12/01/2017 - NA	○							5	○	○	3.7 / 4.5	90
Runtastic Results: Body Workout Fitness Trainer	W	1763 / 0	FREE	163 /NA	NA - 12/12/2016	●	●	○	○	○	●	●	5	○	○	4.5 / NA	95
C25K® - 5K Running Trainer	RP	NA / 29795	FREE	NA/ 38.53	03/01/2017 - NA	●	●	○	○	○	●	●	7	○	●	NA / 4.6	86
Health Mate – Steps tracker & Life coach	P	758/ NA	FREE	124 /NA	03/08/2016 - NA	●	●	●	○	●	●	●	8	○	○	3.2 / NA	90

**Name**	Type of PA app	Number of ratings (iTunes / GP)	Cost (iTunes / GP)	Size in MB (iTunes / GP)	Last update (iTunes / GP)	Have privacy policy	Available without app download	Available after app download	Short form notice	Other languages	Collects PII	Shares with 3rd party	Total number of BCTs	Expert involvement	Any studies associated with the app	Avg. user rating (1-5 stars) (iTunes / GP)	SUS
One You Couch to 5K	RP	92 / 206	FREE	73.9 / 33.07	01/08/2016 - NA	●	●	●	○	○	●	●	12	○	○	1.8 / 2.4	71
Running, Walking and Biking with Endomondo	TR	3698 / 377971	FREE	131 / 18.24	15/01/2017 - 15/12/2016	●	●	○	○	○	●	●	7	○	●	4.4 / 4.5	86
Map My Ride – GPS Cycling & Route Tracker	TR	10197/ 78204	FREE	161 / 68.09	21/12/2016 - 22/12/2016	●	●	●	○	○	●	●	8	○	●	4.2 / 4.4	85
Interval Timer	IT	NA / 5827	FREE	NA/ 4.82	NA - 24/04/2016	○							1	○	○	NA / 4.7	83
5K Run - Couch to 5K	RP	NA / 1615	FREE	NA/ 26.82	NA - 29/12/2016	●	●	○	○	○	●	●	6	○	○	NA / 4.3	91
7 Minutes Workout – Women Fitness Exercise Trainer	W	110 / NA	FREE	92.7 /NA	18/09/2016 - NA	○							8	○	○	4.3 / NA	94

**Name**	Type of PA app	Number of ratings (iTunes / GP)	Cost (iTunes / GP)	Size in MB (iTunes / GP)	Last update (iTunes / GP)	Have privacy policy	Available without app download	Available after app download	Short form notice	Other languages	Collects PII	Shares with 3rd party	Total number of BCTs	Expert involvement	Any studies associated with the app	Avg. user rating (1-5 stars) (iTunes / GP)	SUS
Seconds – Interval Timer	IT	633/ NA	FREE	11.2 /NA	23/11/2015 - NA	●	●	○	○	○	●	●	1	○	○	4.6 / NA	69
Running Distance Tracker +	TR	NA / 4116	FREE	NA/ 12.15	NA - 11/01/2017	●	●	○	NA	○	○	○	3	○	○	NA / 4.6	91
Freeletics Bodyweight – Workout	W	602 / 77767	FREE	70.6 / 38.97	12/01/2017 - 11/01/2017	●	●	○	○	●	●	●	11	○	○	4 / 4.5	84
Couch to 10K Running Trainer	RP	NA / 5856	FREE	NA/ 40.06	NA - 16/01/2017	●	●	○	○	○	●	●	7	○	○	NA / 4.6	86
FitNotes - Gym Workout Log	W	NA / 12698	FREE	NA/ 1.88MB	NA - 30/11/2016	○							6	○	○	NA / 4.5	65
Belly Fat Exercises	W	NA / 1475	FREE	NA/ 3.93MB	NA - 30/07/2016	○							1	○	○	NA / 3.9	73

Name	Type of PA app	Number of ratings (iTunes / GP)	Cost (iTunes / GP)	Size in MB (iTunes / GP)	Last update (iTunes / GP)	Have privacy policy	Available without app download	Available after app download	Short form notice	Other languages	Collects PII	Shares with 3rd party	Total number of BCTs	Expert involvement	Any studies associated with the app	Avg. user rating (1-5 stars) (iTunes / GP)	SUS
Belly Fat Workout FREE – 10 Minute Ab Exercises	W	672 / NA	FREE	74.3 /NA	18/11/2016 - NA	●	●	○	NA	○	NA	NA	5	●	○	4.3 / NA	75
Movesum – Step counter by Lifesum	P	14 / NA	FREE	43.6 /NA	24/09/2016 - NA	●	●	○	○	○	●		4	○	○	1.9 / NA	99
7 Minute Workout Challenge by Fitness Guide Inc	W	1247/ 3697	2.29/2.48	102 / 43.88	20/04/2015 - 23/02/2015	○							11	●	○	4.7 / 4.4	100
Adrian James 6 Pack Abs Workout	W	801/ NA	0.79 / NA	73.5 /NA	20/09/2016 - NA	●	●	○	○	○	●	○	9	●	○	4.6 / NA	99
Full Fitness : Exercise Workout Trainer	W	1455 / NA	2.29 / NA	105 /NA	20/12/2016 - NA	○							9	○	○	4.4 / NA	61
Runtastic PRO Running and Workout Tracker	TR	3845 / 177277	3.99 / 4.99	143 / 33.3	17/01/2017 - 22/12/2016	●	●	○	○	○	●	●	9	○	○	4.6 / 4.5	95

Name	Type of PA app	Number of ratings (iTunes / GP)	Cost (iTunes / GP)	Size in MB (iTunes / GP)	Last update (iTunes / GP)	Have privacy policy	Available without app download	Available after app download	Short form notice	Other languages	Collects PII	Shares with 3rd party	Total number of BCTs	Expert involvement	Any studies associated with the app	Avg. user rating (1-5 stars) (iTunes / GP)	SUS
Couch to 5K® - Running App and Training Coach	RP	127 / 9207	1.49 / 1.67	97.2 / 15.4	30/11/2016 - 18/11/2016	●	●	●	○	○	●	●	9	○	○	4.3 / 4.6	78
Adrian James High Intensity Interval Training	W	77/ NA	0.79 / NA	82.2 /NA	20/09/2016 - NA	●	●	○	○	○	●	○	7	●	○	4.8 / NA	99
Running for Weight Loss PRO	TR	3825 / NA	1.49 / NA	104 /NA	16/12/2016 - NA	●	●	○	○	○	●	●	10	○	○	4.6 / NA	91
Fitness Trainer FULL version	W	NA / 2876	NA / 0.99	NA/ 52.36	NA - 29/12/2016	○							7	○	○	NA / 4.7	78
Instant Fitness : 600+ exercises, 100+ workouts...	W	79 / NA	2.29 / NA	80.2 /NA	30/05/2014 - NA	●	●	○	○	○	●	●	7	○	○	4.3 / NA	89
Push ups 0 to 100: push ups challenge trainer pro	W	106 / NA	1.49 / NA	65.6 /NA	06/10/2016 - NA	●	●	○	NA	○	○	○	9	○	○	4.8 / NA	99

**Name**	Type of PA app	Number of ratings (iTunes / GP)	Cost (iTunes / GP)	Size in MB (iTunes / GP)	Last update (iTunes / GP)	Have privacy policy	Available without app download	Available after app download	Short form notice	Other languages	Collects PII	Shares with 3rd party	Total number of BCTs	Expert involvement	Any studies associated with the app	Avg. user rating (1-5 stars) (iTunes / GP)	SUS
Couch to 5K Runner, 0 to 5K run training	RP	1678 / NA	2.29 / NA	84.3 /NA	04/10/2016 - NA	●	●	○	NA	○	○	○	10	○	○	4.9 / NA	99
Footsteps – Pedometer	P	221 / NA	2.29 / NA	9.3 /NA	13/12/2015 - NA	●	●	○	NA	○	○	○	3	○	○	3.4 / NA	61
iMuscle 2	W	56 / 2128	2.79 / 1.89	376 / 145	08/12/2014 - 22/07/2016	●	●	○	○	○	●	●	10	●	○	4.8 / 4.1	66
10K Running Trainer Pro	RP	NA / 570	NA / 2.49	NA/ 27.64	NA - 09/01/2017	●	●	○	○	○	●	●	8	○	○	NA / 4.5	88
Police Fitness – Bleep Test	W	20 / NA	0.79 / NA	30.6 /NA	27/01/2017 - NA	○							3	○	○	4 / NA	80
Marathon Trainer - 26.2 42K	RP	NA / 136	NA / 8.49	NA/ 27.66	NA - 10/01/2017	●	●	○	○	○	●	●	8	○	○	NA / 4.4	88

**Name**	Type of PA app	Number of ratings (iTunes / GP)	Cost (iTunes / GP)	Size in MB (iTunes / GP)	Last update (iTunes / GP)	Have privacy policy	Available without app download	Available after app download	Short form notice	Other languages	Collects PII	Shares with 3rd party	Total number of BCTs	Expert involvement	Any studies associated with the app	Avg. user rating (1-5 stars) (iTunes / GP)	SUS
MapMyFitness+ Workout Trainer	W	NA / 1409	NA / 2.30	NA/ 88.23	NA - 31/01/2017	●	●	●	○	●	●	●	10	○	●	NA / 4.4	93
5K to 10K	RP	16 / 378	NA 2.29/ 2.32	79.4 / 12.92	30/12/2015 - 25/05/2016	●	●	●	○	○	●	●	10	○	○	3.9 / 4.3	88
7 Minute Workout Pro	W	NA / 1521	NA / 2.39	NA/ 28.2	NA - 26/09/2016	●	●	●	NA	○	○	○	13	○	○	NA / 4.6	85
Chloe Madeley Weights 4 Women	W	11 / 7	NA2.99/ 2.99	102 / 60.19	03/05/2016 - 04/05/2016	●	●	●	○	○	●	●	2	●	○	2.5 / 3.9	84
10K Pacer: Run pace training. Run faster	RP	12 / NA	NA2.99/ NA	65.3 /NA	08/10/2016 - NA	●	●	○	NA	○	○	○	10	○	○	4.8 / NA	99
Starting Strength Official	W	NA / 391	NA/ 8.99	NA/ 55.58	NA - 15/12/2016	○							9	●	○	NA / 4.6	58

**Name**	Type of PA app	Number of ratings (iTunes / GP)	Cost (iTunes / GP)	Size in MB (iTunes / GP)	Last update (iTunes / GP)	Have privacy policy	Available without app download	Available after app download	Short form notice	Other languages	Collects PII	Shares with 3rd party	Total number of BCTs	Expert involvement	Any studies associated with the app	Avg. user rating (1-5 stars) (iTunes / GP)	SUS
Thor Fitness: 60 Day Bodyweight Workout Routine	W	17 / NA	0.79 / NA	242 /NA	10/06/2015 - NA	○							8	○	○	4.4 / N	69
Half Marathon Trainer 13.1 21K	RP	47 / NA	7.99 / NA	90.7 /NA	06/01/2017 - NA	●	●	○	○	○	●	●	8	○	●	4.3 / NA	85
Yoga Break	W	not displayed / NA	2.29 / NA	98.5 /NA	09/02/2016 - NA	○							5	○	○	not displayed / NA	93
PDC Pole Dance Syllabus	W	NA / 15	NA / 2.99	NA/ 0.97	NA - 14/08/2016	●	●	○	NA	○	●	○	1	●	○	NA / 3.5	83
MMA Spartan Workouts Pro	W	NA / 1920	NA / 2.99	NA/ 44.07	NA - 21/09/2016	○							6	○	○	NA / 4.6	90
Get Running (Coach to 5K)	RP	2670 / NA	2.29 / NA	24.2/NA	16/10/2013 - NA	○							5	○	●	4.7 / NA	88

Name	Type of PA app	Number of ratings (iTunes / GP)	Cost (iTunes / GP)	Size in MB (iTunes / GP)	Last update (iTunes / GP)	Have privacy policy	Available without app download	Available after app download	Short form notice	Other languages	Collects PII	Shares with 3rd party	Total number of BCTs	Expert involvement	Any studies associated with the app	Avg. user rating (1-5 stars) (iTunes / GP)	SUS
WalkJogRun GPS Running Routes	TR	935 / NA	3.99 / NA	44.8 /NA	04/10/2016 - NA	○							5	○	○	3.9 / NA	71
Runtastic Road Bike PRO	TR	NA / 14840	NA / 4.99	104 / 22.35	NA - 01/03/2016	●	●	○	○	○	●	●	7	○	○	NA / 4.4	91
Runtastic Mountain Bike PRO GPS Biking Computer,	TR	232/ 14955	3.99 / 4.99	59.2 / 21.41	12/09/2016 - 01/03/2016	●	●	○	○	○	●	●	6	○	○	4.8 / 4.5	94
Chloe Madeley 15 minute fat loss workout	W	56 / NA	2.99 / NA	85.4 / 9.64	29/07/2016 - NA	○							3	●	○	3.4 / NA	90
CARROT Fit – 7 Minute Workout, Step Counter Weight Tracker	W	263 / NA	2.49 / NA	164 / 70.02	17/11/2016 - NA	●	●	●	○	○	○	NS	11	○	○	4.7 / NA	94

Notes: The assessment of User involvement and Organizational affiliation is not displayed in this table as no apps reported consulting users in the app development; 1 app, One You Couch to 5K, was indicated as having governmental affiliation; App types: IT; Interval timer-type app; P; Pedometer - type app; RP, Running programme-type app; TR; Tracking of running-type app; W, Workout-type app; MB, megabites; NA, not applicable; NS, not specified; PPI, Personally Identifiable Information; ●, Yes; ○, No.
