# Supplementary figures and images for: Quality of Publicly Available Physical Activity Apps: Review and Content Analysis
Source: JMIR Mhealth Uhealth. 2018 Mar 21;6(3):e53. doi: 10.2196/mhealth.9069 (PMC5885062; doi:10.2196/mhealth.9069)

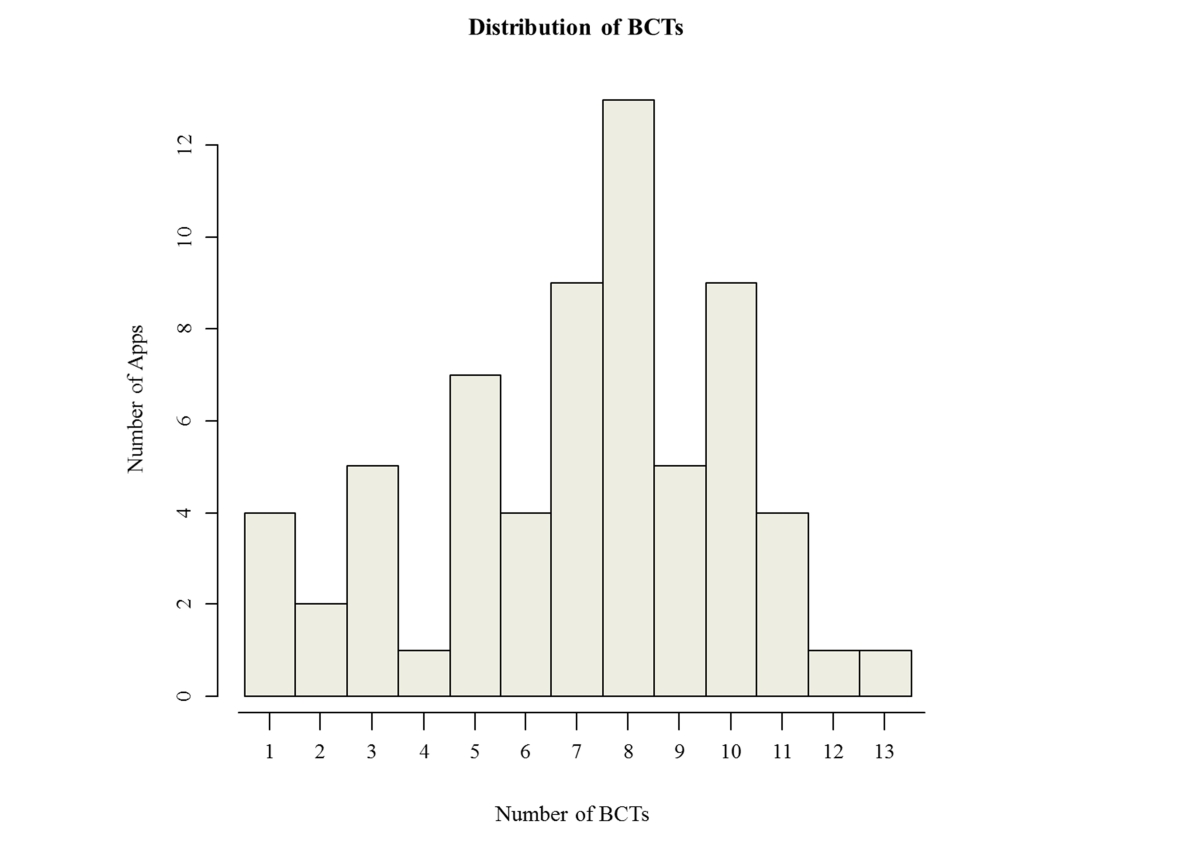

Supplement: Multimedia Appendix 3 [file mhealth_v6i3e53_app3.jpg]

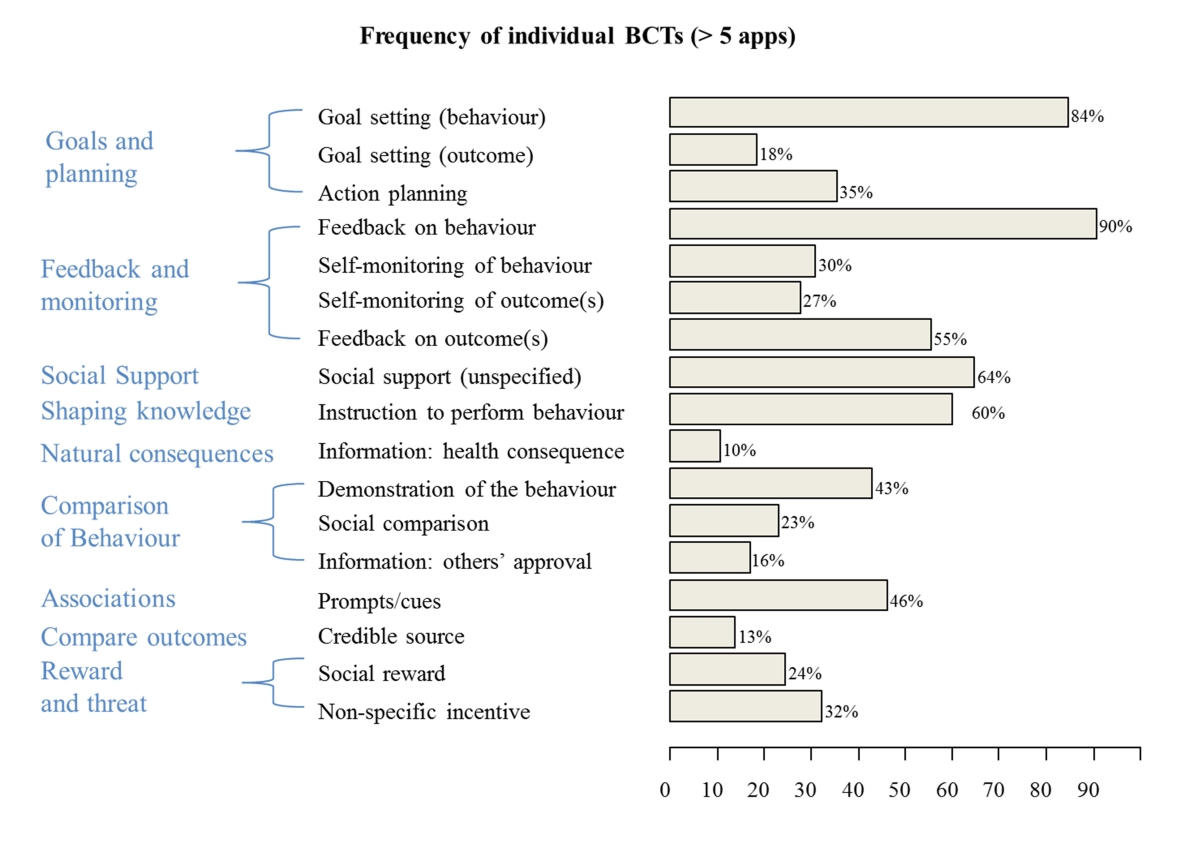

Supplement: Multimedia Appendix 4 [file mhealth_v6i3e53_app4.jpg]

## SUS

Average of both reviewers

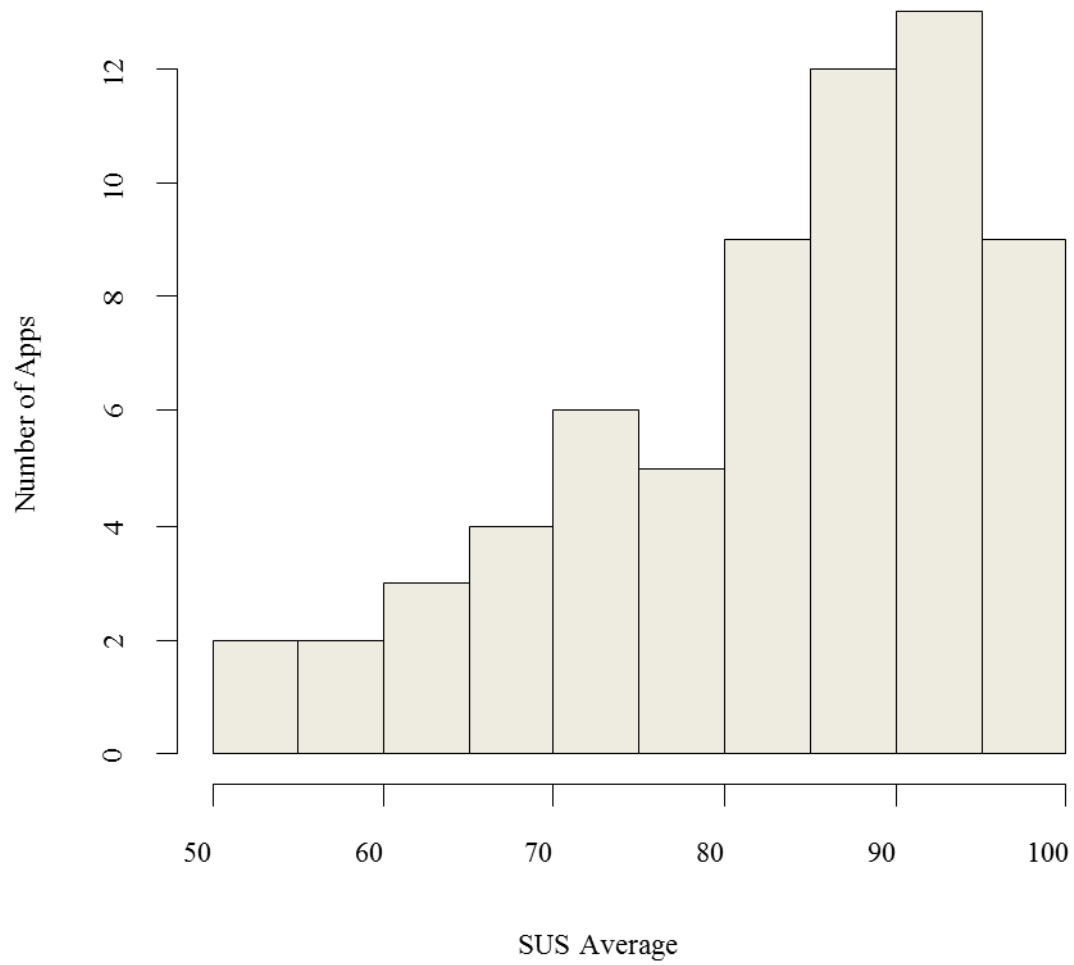

Supplement: Multimedia Appendix 5 [file mhealth_v6i3e53_app5.pdf]
